# Supplementary material for: Methylation-Sensitive Expression of a DNA Demethylase Gene Serves As an Epigenetic Rheostat
Source: PLoS Genet. 2015 Mar 31;11(3):e1005142. doi: 10.1371/journal.pgen.1005142 (PMC4380477; doi:10.1371/journal.pgen.1005142)
Supplement: S1 Text — (DOC) [file pgen.1005142.s001.doc]

**S1 Text: Supplemental Materials and Methods**

**Arabidopsis mutants**

The mutants used for this study were obtained from the Arabidopsis Biological Resource Center (ABRC) [60], Ohio State University, unless otherwise stated.

*ago4:* CS9927

*ago6*: CS66096

*ago4; ago6:* CS66095

*cmt2*: CS849188

*cmt3*: CS3665

*dcl3:* CS16390

*ddm1*: *ddm1-5* [61]

*dms3:* SALK_068723C

*drm1; drm2:* CS6366

*hda6:* CS66153 *(axe1-5)*

*ibm1*: SALK_035608C

*kyp:* SALK_044606C

*met1: met1-6* [50]

*nrpd1a*: SALK_128428

*nrpe1:* SALK_029919C

*rdr2-1:* CS66076

*rdr6:* CS24285

*ros1-2:* Obtained from Jian-Kang Zhu [32]

*ros1-7:* Obtained from the TILLING project [33]

*shh1:* SALK_074540C

*spt5l:* SALK_001254C

*vim1; vim2; vim3:* Obtained from Eric Richards [62]

*mop1-1* (maize): Obtained from Nathan Springer [63]

**Primers used in this study** (5’🡪3’)

**RT-qPCR:**

*ROS1* F: CAGGCTTGCTTTTGGAAAGGGTACG

*ROS1* R: GTGCTCTCTCACTCTTAACCATAAGCT

*DML2* F: CGGGAAGAGGAATCACAGACT

*DML2* R: GGACGTCGATAGGGTTTATGCT

*DML3* F: CGTAGGGAGTTGTGTAAGGGA

*DML3* R: GCAAAGTTCAATCCGTCTTGTGT

*IBM1* F: TGCTGTCCTGTGTCTCAGGTTG

*IBM1* R: ACCGCGTCAGATAGAAGTTCTGG

AT1G58050 F: CCATTCTACTTTTTGGCGGCT

AT1G58050 R: TCAATGGTAACTGATCCACTCTGATG

*AlROS1a/b* F: TTGCTATTTGGACGCCAGGTGAG

*AlROS1a* R: ATACACTTACTTACAGCCGGTTG

*AlROS1b* R: ATACACTTGCTCACAGTTGGTTG

Al[315392](http://www.phytozome.net/genePage.php?search=1&searchText=transcriptid%3A16036588&crown&method=0&detail=1)* F: CCATTCTACTTTTTGGCGGCT

Al[315392](http://www.phytozome.net/genePage.php?search=1&searchText=transcriptid%3A16036588&crown&method=0&detail=1)* R: TCAATGGTTACTGATCCACTCTGATG

**A. lyrata* homolog to AT1G58050

*DNG101* F: CCAGATGATCCCTGTCCATATCTTC

*DNG101* R: GGCATCGATCGATTGTGCAGTTTC

*DNG103* F: CCATGCTGTGACCCTCAAATG

*DNG103* R: CTCTGCAGTACAATTGTGGCAC

*ZmEF1α* F: TGGGCCTACTGGTCTTACTACTGA

*ZmEF1α* R: ACATACCCACGCTTCAGATCCT

**Bisulfite Sequencing:**

*AtROS1* 5' end top strand fragment 1a F: GAYTAAAAYATTTGGAATGATYAAAAAYGAAAG

*AtROS1* 5' end top strand fragment 1a R: TTGTTTTCTACAAAATCTCCTARACTAT

*AtROS1* 5' end bottom strand fragment 1b F: CAACTARCCTAATAATCACTCTACTACACT

*AtROS1* 5' end bottom strand fragment 1b R: TAGAYTATGGGAAAGATGATTTAAAAAG

*AtROS1* 5' end top strand fragment 1c F: GGAGATTTTGTAGAAAAGAATYATT

*AtROS1* 5' end top strand fragment 1c R: TCACTRATRCTTCRTTTCTTCTCTT

*AtROS1* 5' end bottom strand fragment 1d F: CTTTTTAAATCATCTTTCCCATARTCTA

*AtROS1* 5' end bottom strand fragment 1d R: GTAGAATYAATGGTTATGGTGGTG

*AtROS1* coding region fragment 2a F: CACAAACCTTTCCTCCAATTRACTRCTAT

*AtROS1* coding region fragment 2a R: GATTYYAAGAGAAGAAAYGAAGYATYAGTG

*AtROS1* coding region fragment 2b F: CACAAACCTTTCCTCCAATTRACTRCTAT

*AtROS1* coding region fragment 2b R: GATYTGYYYATAYAYGGTGGAGGAT

*AtROS1* coding region fragment 3 F: CATCCCRCRACTCTTRATTRTTTCARCAAC

*AtROS1* coding region fragment 3 R: GAGYAGGATYAAGYTYAGAGATYGAYTTAG

*AtROS1* coding region fragment 4 F: CRCCTCCTCTATRTCARCTATTRATACTTC

*AtROS1* coding region fragment 4 R: GYYAGTGYGTTTGYAAGGTGYTAYAAYATG

*AtROS1* coding region fragment 5a F: TTGTGGYAGTTGGAAAAGAGAGAAYYTG

*AtROS1* coding region fragment 5a R: CTTACCTCATTTACTTRAAARTACRTTCC

*AtROS1* coding region fragment 5b F: GGAAYGTAYTTTYAAGTAAATGAGGTAAG

*AtROS1* coding region fragment 5b R: CCACRTACACATACRTACCCTACATA

*AlROS1a* 5' end top strand F: TGTTAAAGAAAAGGATAGAAYATGTGTG

*AlROS1a* 5' end top strand R: CCTAATAATCACCCTATAACTTCCT

*AlROS1a* 5' end bottom strand F: TCTTTCTCTAACTTTCATARCCRTTT

*AlROS1a* 5' end bottom strand R: AAGAATTGTAAGGGGAYTAGYYTAAT

*AlROS1a* exons 19-20 F: TTGYTAGTGYGTTTGYAAGGTGYTA

*AlROS1a* exons 19-20 R: CATCCTCTATRTCARCTATTRATACTT

*AlROS1b* exons 18-19 F: GTATGTGAAGTAAYTGGATAAGGAYAYATG

*AlROS1b* exons 18-19 R: CTTACCTCATTTACTTRAAARTACRTTCC

**5' RACE:**

*ROS1* cDNA synthesis primer: CTCACAGTCACCCGCGTATCA

*ROS1* RACE outer nested PCR: GACTGCTATGATATTGATCCTCCAC

*ROS1* RACE inner nested PCR: GCTTTCTTCTCTCCTCTGTTTCTCCAT

**Transgene construction:**

Inverted repeat F: CACCGTTAGTTCATATAATTTTAAATAGTTACGT

Inverted repeat R: AGGGCGAAAGTTCGTTTGGTTG

**Supplemental References**

60. Alonso JM, Stepanova AN, Leisse TJ, Kim CJ, Chen H, Shinn P, et al. Genome-Wide Insertional Mutagenesis of Arabidopsis thaliana. Science. 2003;301: 653–657. doi:10.1126/science.1086391

61. Jeddeloh JA, Stokes TL, Richards EJ. Maintenance of genomic methylation requires a SWI2/SNF2-like protein. Nat Genet. 1999;22: 94–97. doi:10.1038/8803.

62. Woo HR, Dittmer TA, Richards EJ. Three SRA-Domain Methylcytosine-Binding Proteins Cooperate to Maintain Global CpG Methylation and Epigenetic Silencing in Arabidopsis. PLoS Genet. 2008;4: e1000156. doi:10.1371/journal.pgen.1000156.

63. Alleman M, Sidorenko L, McGinnis K, Seshadri V, Dorweiler JE, et al. An RNA-dependent RNA polymerase is required for paramutation in maize. Nature. 2006;442: 295–298. doi:10.1038/nature04884.
