# Supplementary figures and images for: Methylation-Sensitive Expression of a DNA Demethylase Gene Serves As an Epigenetic Rheostat
Source: PLoS Genet. 2015 Mar 31;11(3):e1005142. doi: 10.1371/journal.pgen.1005142 (PMC4380477; doi:10.1371/journal.pgen.1005142)

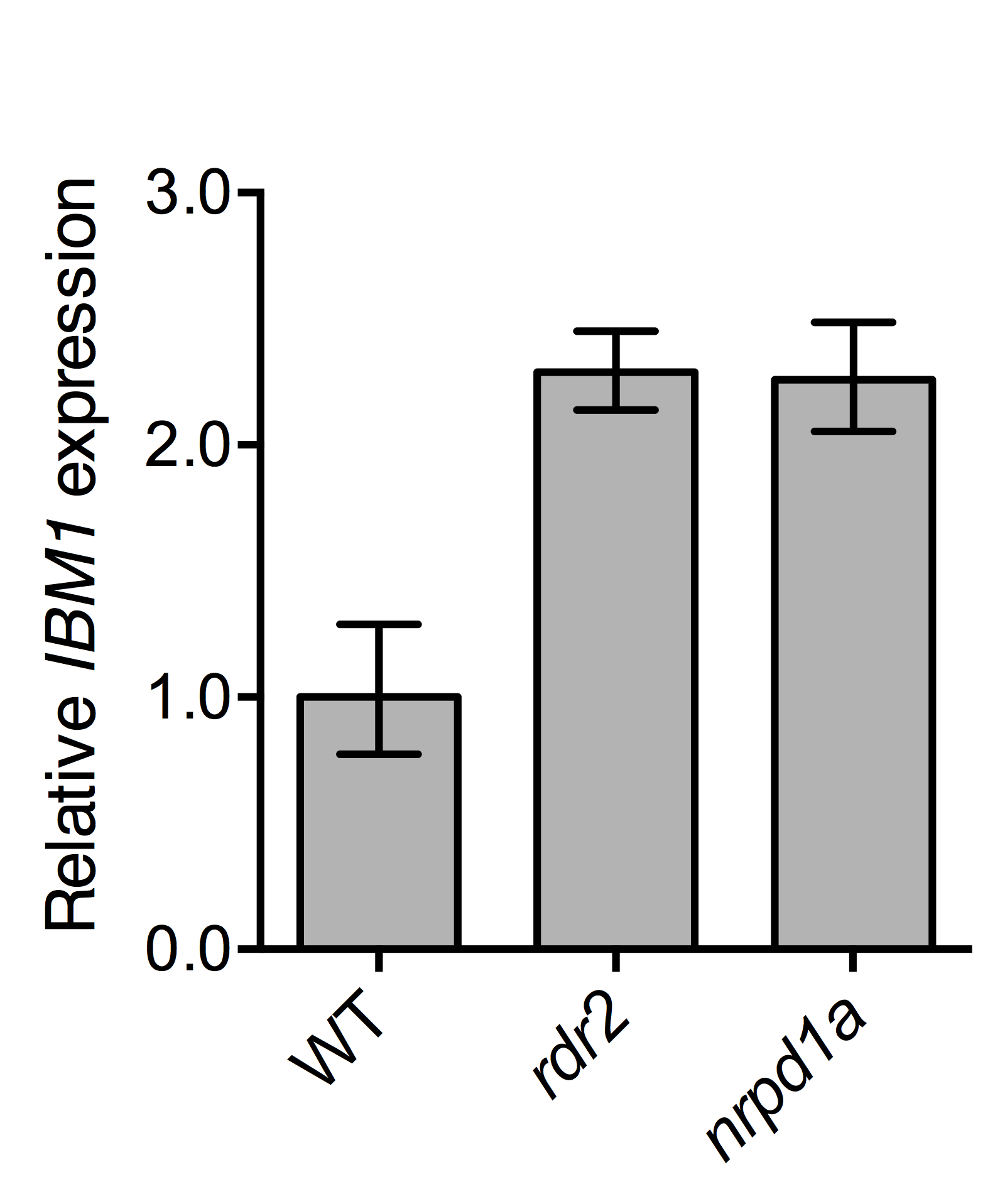

Supplement: S2 Fig — The level of expression of the long transcript isoform of IBM1 was measured in seedlings of two RdDM mutants by RT-qPCR. In both rdr2 and nrpd1a mutants, IBM1 transcript abundance was higher than in WT (Col-0) at p = <0.05. Data represented as mean, error bars represent standard deviation. (TIFF) [file pgen.1005142.s003.tiff]

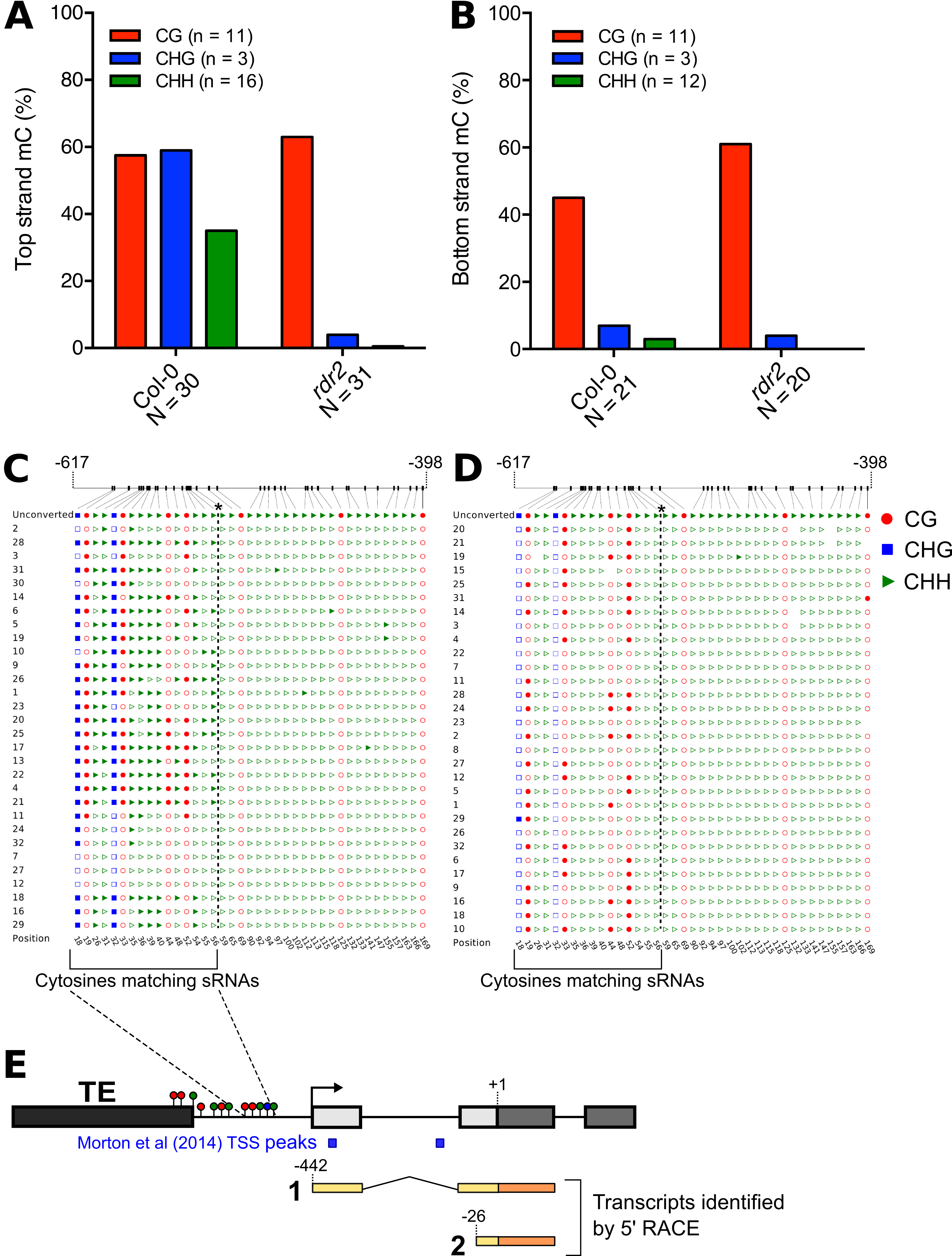

Supplement: S3 Fig — (A-B) Methylation levels of the top and bottom strand of a 228 bp region upstream of the ROS1 transcription start site were quantified using BS-PCR on DNA from wild-type Col-0 and rdr2 mutants. Percentage methylation was calculated from the first to last methylated cytosine. N represents the number of independent clones sequenced; n represents the number of cytosines counted in the region. (C) and (D) show a portion of the cytosines analyzed from the top strand of Col-0 and rdr2, respectively. Filled/unfilled shapes represent methylated/unmethylated cytosines respectively. *; dotted line indicates the 3'-most position from which methylation was quantified. The quantified region continues beyond what is shown at the 5' end. (E) The transcription start sites of ROS1 in WT were determined using 5' RACE. The positions of transcriptional start sites identified by 5’ RACE and in a genome-wide profiling study [59] are shown relative to the region of DNA methylation. (TIFF) [file pgen.1005142.s004.tiff]

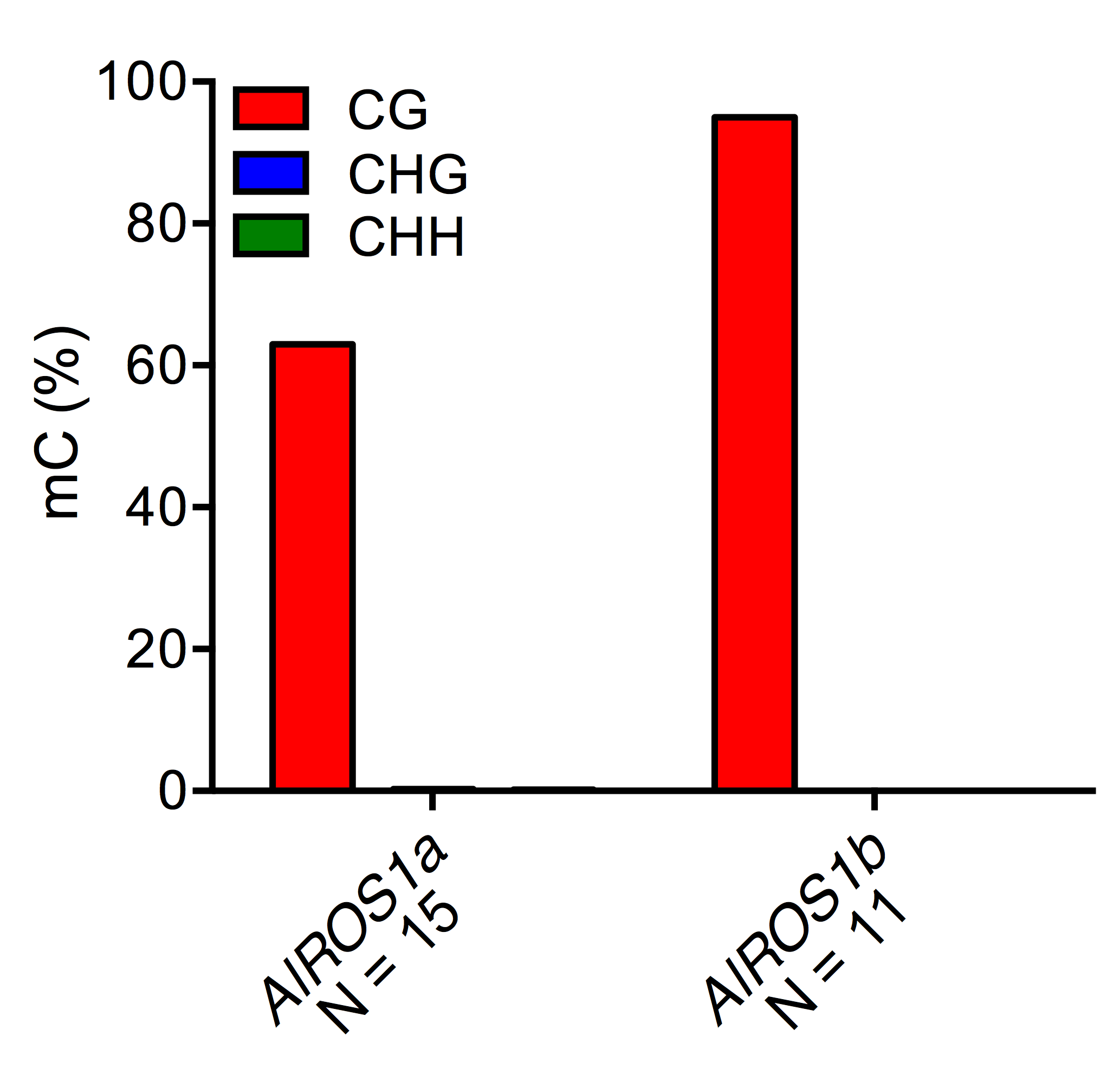

Supplement: S4 Fig — DNA methylation in exons 19–20 of AlROS1a and exons 18–19 of AlROS1b (which share homology with AtROS1 exons 17–18) was measured using BS-PCR on leaf DNA. Percentage methylation in each sequence context was calculated from the first to the last methylated cytosine. 6 CG, 2 CHG and 32 CHH sites were counted for AlROS1a. 7 CG, 11 CHG and 31 CHH sites were counted for AlROS1b. (TIFF) [file pgen.1005142.s005.tiff]

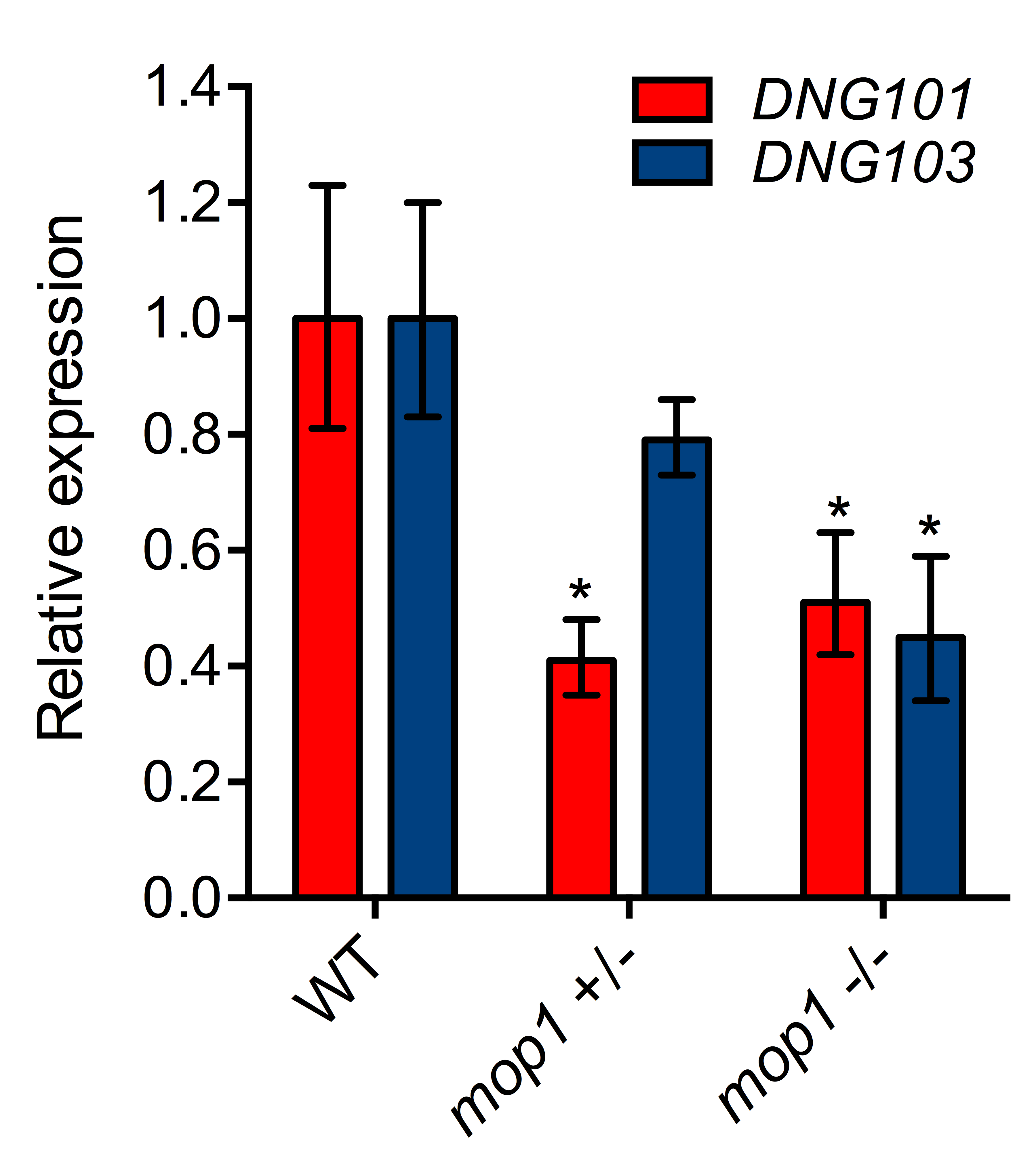

Supplement: S5 Fig — Transcript abundance of the 5-methylcytosine DNA glycosylases DNG101 and DNG103 was measured in maize plants segregating for a mutation in Mop1, a homolog of Arabidopsis RDR2, using RT-qPCR. WT is the inbred strain B73. Data are represented as mean, error bars represent standard deviation. *p = <0.01, two-tailed t-test. (TIFF) [file pgen.1005142.s006.tiff]

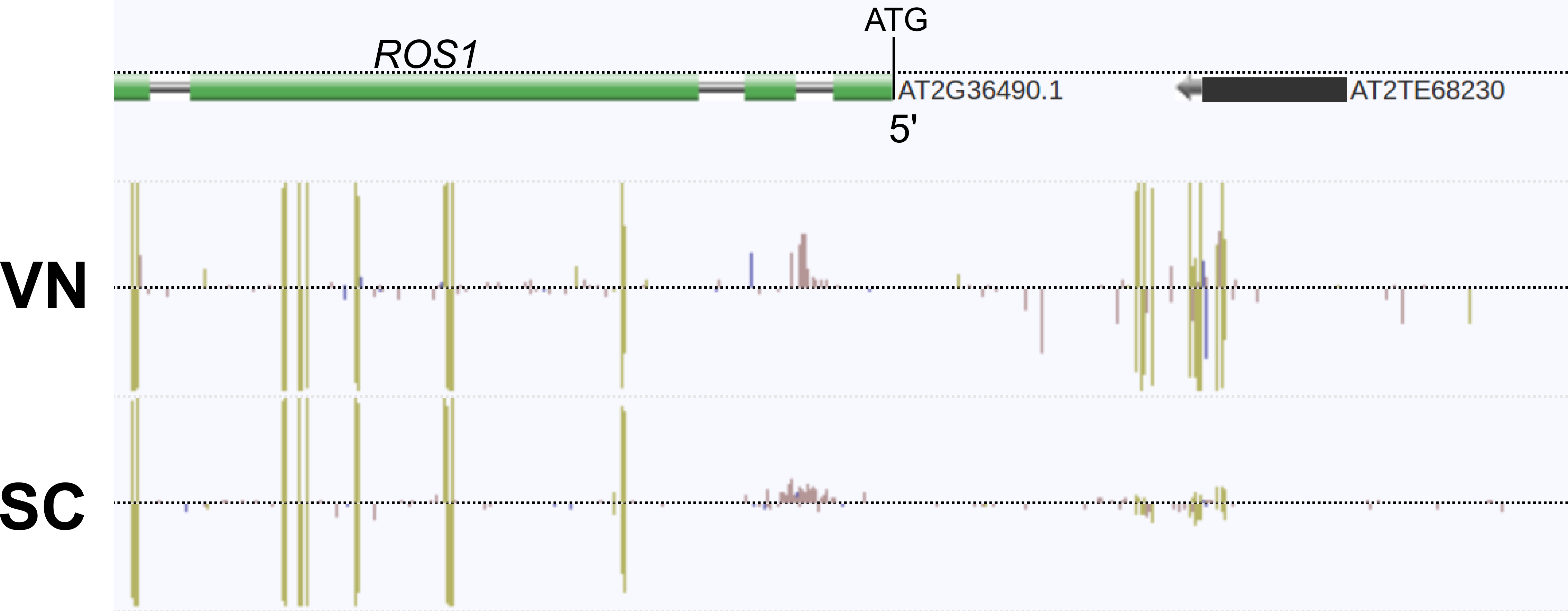

Supplement: S6 Fig — A snapshot of genome-wide methylation data from Arabidopsis pollen [43], showing the 5' region of ROS1. CG, CHG and CHH methylation are denoted by green, blue and red lines, respectively. VN = vegetative nucleus. SC = sperm cells. (TIFF) [file pgen.1005142.s007.tiff]
